# Complete Genomic Sequence of Issyk-Kul Virus

**DOI:** 10.1128/genomeA.00662-15

**Published:** 2015-07-02

**Authors:** Barry Atkinson, Denise A. Marston, Richard J. Ellis, Anthony R. Fooks, Roger Hewson

**Affiliations:** a Microbiology Services Division, Public Health England, Wilts, United Kingdom; b Wildlife Zoonoses & Vector-Borne Diseases Research Group, Animal and Plant Health Agency (APHA), Surrey, United Kingdom; c Specialist Scientific Support Department, APHA, Surrey, United Kingdom; d National Consortium for Zoonosis Research, University of Liverpool, Liverpool, United Kingdom; e Department of Clinical Infection, Microbiology and Immunology, University of Liverpool, Liverpool, United Kingdom; f National Institute for Health Research, Health Protection Research Unit in Emerging and Zoonotic Infections, Liverpool, United Kingdom

## Abstract

Issyk-Kul virus (ISKV) is an ungrouped virus tentatively assigned to the *Bunyaviridae* family and is associated with an acute febrile illness in several central Asian countries. Using next-generation sequencing technologies, we report here the full-genome sequence for this novel unclassified arboviral pathogen circulating in central Asia.

## GENOME ANNOUNCEMENT

Issyk-Kul virus (ISKV) was first isolated in 1970 from a *Nyctalus noctula* bat trapped near Lake Issyk-Kul, Kyrgyzstan; the virus was subsequently identified in Tajikistan and Kazakhstan, with sporadic outbreaks of human disease reported in all 3 countries (1, 2). Clinical symptoms include fever (39 to 41°C), headache, myalgia, and nausea. Fatal outcomes are uncommon, although convalescence may take up to 6 weeks (1, 3). ISKV is likely to have a reservoir in both bats and ticks, with transmission to humans being associated with tick bites, exposure to bat urine/feces, or a possible involvement of mites (4). The virus was tentatively assigned to the *Bunyaviridae* family based on electron microscopy; however, to date, no confirmatory data have been published in English.

The LEIV-315K strain of ISKV was propagated in a suckling mouse model. TRIzol-extracted viral RNA was depleted of host genomic DNA using RNase-free DNase (Qiagen, United Kingdom), and host rRNA was depleted using Terminator 5′-phosphate-dependent exonuclease (Epicentre Biotechnologies), as described previously (5, 6). The depleted RNA was quantified using RiboGreen (Life Technologies). Double-stranded (ds) cDNA was synthesized, using a random-primed method, from 50 ng of depleted RNA using the cDNA synthesis system (Roche), according to the manufacturer’s instructions. ds-cDNA was purified using AMPure XP magnetic beads (Beckman Coulter), with 1 ng used as input for the Nextera XT DNA sample preparation kit (Illumina). The sequencing library was prepared according to the manufacturer’s instructions and sequenced on an Illumina MiSeq instrument with 2 × 150-bp paired-end reads, according to standard Illumina protocols. Sequencing data (27,029,078 reads) were processed to remove host genome by mapping to the mouse reference using BWA version 0.7.5a-r405 (7) and then by *de novo* assembly of unmapped reads (700,000 reads; 2.59%) using Velvet version 1.2.03 (8), with a *k*-mer value of 141. Of the 12 contigs obtained, 9 were identified as host using BLAST analysis and therefore removed from further analysis. The remaining 3 contigs (125,963 reads; 0.46%) were identified as viral with near-complete coverage of S, M, and L segments. Initial data identified the virus as belonging to the *Nairovirus* genus; unresolved terminal regions were confirmed using bespoke pan-nairovirus terminal region primers in combination with internal primers based on our next-generation sequencing (NGS) data. PCR amplicons were Sanger sequenced and aligned with the NGS *de novo* assembled sequences, resulting in complete S, M, and L segments. The NGS data were remapped against these reference sequences using previously described methods (9). A total of 249,869 reads were mapped (0.92% of total reads) as follows: S segment, 47,082 reads; M segment, 103,245 reads; and L segment, 99,542 reads, with average read depths of 3,522×, 2,620×, and 1,099×, respectively. The sequence data for ISKV described here align with independent ISKV virus genome data published during our investigations (GenBank accession numbers KF892055 to KF892057), with only 5 point mutations across all 3 segments. Importantly, our results confirm the terminal regions for all 3 segments that were not resolved by the initial submissions. The characterization of the genome will help improve our understanding of this human disease and provide data for the development of molecular diagnostics.

### Nucleotide sequence accession numbers. 

The complete genomic sequence of ISKV has been deposited in GenBank under the accession numbers KR709219 to KR709221.
